# Parents’ differential susceptibility to a “micro” parenting intervention: Rationale and study protocol for a randomized controlled microtrial

**DOI:** 10.1371/journal.pone.0282207

**Published:** 2023-03-22

**Authors:** Rabia R. Chhangur, Jay Belsky

**Affiliations:** 1 Department of Developmental Psychology, Tilburg University, Tilburg, The Netherlands; 2 Department of Human Ecology, University of California Davis, Davis, California, United States of America; St John’s University, UNITED STATES

## Abstract

**Background:**

Given evidence that parenting can influence children’s development, parenting interventions are often the strategy of choice when it comes to treating children’s disruptive behavior problems—or preventing problems from developing in the first place. What remains under appreciated, however, is that some parents appear to be more responsive to interventions to foster skilled parenting than others. Notable in this regard is the ever-increasing observational and, perhaps more importantly, experimental evidence indicating that some children prove more susceptible to parenting interventions than others. So, while the experimental evidence clearly indicates that “susceptibility factors” which children carry seem to affect their likelihood of benefiting from a parenting intervention (and other environmental influences), what remains unclear is why the parenting interventions in question prove more effective in changing the behavior of some parents more than others. Could it be as a result of their own parental characteristics?

**Objective:**

The Parfective Microtrial in a randomized controlled microtrial, in which we focus not just on parental (and child) responsiveness but also on an underlying physiological mechanism hypothesized to contribute to heightened susceptibility to parenting interventions.

**Methods:**

Participants are 120 families, with children aged 4–5 years, recruited from the community. Of these, 60 are randomly assigned to the “micro” intervention condition (i.e., immediate positive parenting feedback) and 60 families to the care-as-usual control condition. Assessments in both conditions will be conducted at baseline (pretest), after 2 weeks (posttest), and after 4 weeks (follow-up). Primary outcomes are the hypothesized moderating effects of physiology on the anticipated “micro” intervention effect (i.e., decrease in negative parenting behavior and/or increase in positive parenting behavior). Secondary outcomes are the observed (changes in) child behavior in response to the parenting intervention, such that those parents and children—in the same family—who manifest these physiological attributes will prove most susceptible to the beneficial effects of the intervention.

**Trial registration:**

This study protocol is registered at ClinicalTrials.gov (NCT05539170).

## Introduction

Parenting is widely regarded as an important *and* modifiable determinant of children’s problem behavior, one which has thus proven responsive to intervention [1]. While intervention effects are small in magnitude, with even the most successful programs proving effective for only 25% of enrolled families [2], it remains under appreciated that some parents appear to be more responsive to interventions to foster skilled parenting than others. Thus, what remains unclear is *why* some parents prove more susceptible to effects of intervention on parenting than do others. Notable in this regard is the ever-increasing observational and, perhaps more importantly, experimental evidence indicating, respectively, that *(a)* some *children* prove more susceptible to (observed) parenting and parenting interventions than others because of their own “biological sensitivity”, be they behavioral/temperamental, physiological, and/or genetic in nature; and *(b)* that intervention responsiveness is especially pronounced when parents themselves evince more rather than less change in their parenting in response to the intervention [3, 4]. What remains unclear is why some parents prove more responsive when it comes to changing their parenting in response to interventions than others. We hypothesize that it is due, at least in part, to their own underlying physiological processes which can be measured with physiological signals (e.g., heart rate, skin conductance) by means of wearable technology.

Given that parenting interventions are often the strategy of choice when it comes to treating disruptive behavior problems in children—or preventing problems from developing in the first place—it is perhaps surprising that no experimental intervention research has focused on differential change in parenting behavior in response to intervention or on underlying biological/physiological processes that might account for parents’ differential response to intervention [5]. Some *observational* evidence does indicate, however, that parents’ biological sensitivity affects the extent to which a variety of contextual conditions influence parenting. Consider in this regard evidence showing that low and high quality levels of environmental context predict, respectively, less and more positive parenting, but specifically in the case of parents carrying select plasticity genes [6, 7]. Such evidence raises the possibility that parents’ own characteristics of biological sensitivity—obtained from physiological signals are also associated with their susceptibility to parenting interventions, a possibility we aim to evaluate by using a randomized controlled microtrial approach. Microtrials are defined as powerful randomized experiments, testing effects of brief environmental manipulations designed to suppress specific risk mechanisms or enhance specific protective mechanisms, but not to bring about full treatment or prevention effects on temporally distal outcomes [8, 9]. More specifically, we will use a “micro” intervention that has been proven effective in fostering parents’ positive parenting and children’s positive behavior, by providing immediate positive feedback about one’s competence as a parent [10, 11].

### Physiological-based differential susceptibility

Within the last decades, the differential susceptibility hypothesis [12], a within-person proposition that stipulates that the same individuals who are most vulnerable to adversity may also benefit most from enriched environments, has received much scientific attention over the past decade. The majority of research on this hypothesis has been conducted on children and the parenting they receive. There is repeated indication that children differ in the extent to which they benefit from improved parenting or suffer from dysfunctional parenting due to their individual characteristics [13, 14]. These individual differences “for better and for worse” are believed to be rooted in certain endogenous, biological factors that include *(a)* genes, *(b)* temperamental characteristics, and most importantly for this study, *(c)* physiological endophenotypes (e.g., heart rate variability and skin conductance) (see for example [15, 16]). So, while the experimental evidence clearly indicates that some *children* are likely to benefit from a parenting intervention (and other environmental influences), what remains unclear is why the parenting interventions in question prove more effective in changing the behavior of some parents more than others. Could it be as a result of *parental* physiological endophenotypes? Quite conceivably, then, some children—based on their own physiological characteristics—might change more in response to change in parents’ positive parenting, which itself is a result of parents’ susceptibility to environmental influence.

Endophenotypes are genetically informed, stable bio-behavioral traits that function as mediators between genes and phenotype [17, 18]. They seem to be of relevance to differential susceptibility research because of their role in sensitizing the individual to their environmental exposures [19, 20]. We speculate that negativity and positivity actually reflect a highly sensitive nervous system on which experience registers powerfully [21]—negatively when not regulated but positively when positive valence is elicited—a point of view somewhat related to that biological-sensitivity-to-context proposal that individuals who are highly physiologically reactive to stress manifest the most developmental plasticity [22]. More specifically, physiological endophenotypic signals such as heart rate (HR) and skin conductance (electrodermal activity, EDA) are increasingly being used in clinical practice and child healthcare research as they provide innovative ways to bring neuroscience from the lab to real-life settings [23–25]. Thus, we will rely on a very promising state-of-the-art strategy but *minimally invasive* approach, which makes it possible to evaluate differential parental/child responsiveness to a brief and effective “micro” parenting intervention. Notable in this regard is evidence that parents’ physiological reactivity could indeed account for greater and lesser responsiveness to intervention effects, in that only fathers with higher physiological reactivity showed increased positive parenting after receiving a parenting intervention designed for military families with school-aged children [26]. This suggests that physiological reactivity may be a biomarker that makes some parents more responsive to intervention effects than others [27].

### Microtrial to detect intervention heterogeneity

Randomized controlled trials (RCTs) of full-scale interventions can provide insight into these questions—that is, they utilize experimental designs that can provide evidence of intervention heterogeneity that complement and extend data from non-experimental investigations. In addition, the experimental feature of these intervention studies can greatly increase their power to detect interaction effects as compared to observational-correlational studies [28]. However, most RCTs have only limited capacity to illuminate why some individuals benefit or provide insight as to what plasticity mechanisms might be productively targeted in individuals who do not benefit. Empirically addressing these issues requires explicit focus on mechanisms through which risk and protective factors might exert their effects, including whether these mechanisms involve biological endophenotypes. Although RCTs of full-scale interventions can provide some insight into these issues, their utility is constrained by the considerable cost and effort required to measure biological processes, and by the complex and multifaceted nature of the interventions employed [9].

By contrast, experimental studies designed as randomized controlled microtrials might be better suited for analyzing specific effects of distinct parenting variables on parenting and child outcomes. Recall that microtrials are based on relatively brief and focused environmental manipulations designed to suppress specific risk mechanisms and/or enhance specific protective mechanisms, but not to bring about full treatment or prevention effects in distal outcomes. In particular, a randomized controlled microtrial can test hypotheses about underlying behavioral processes by examining whether certain mechanisms change in the experimental condition, mediating the intervention effect [8]. Indeed, if the underlying plasticity process can be illuminated in a less expensive microtrial is would provide an empirical basis for moving to more extensive RCTs.

### Hypothesis and aim of the study

The Parfective Microtrial is a randomized controlled microtrial informed by a physiologically-based measurement tool to illuminate determinants of variation in response to a ‘micro” parenting intervention, using a community sample. The study examines not just parental (and child) intervention responsiveness, but also an underlying physiological mechanism thought to contribute to heightened susceptibility to parenting interventions. In so doing, this study addresses a widely-appreciated need when it comes to advancing understanding of why some individuals prove more responsive to intervention efforts—the need to illuminate underlying biological mechanisms that instantiate differential susceptibility to interventions. Moreover, it will be the very first to simultaneously focus on parent susceptibility to the intervention and child susceptibility to change in parenting induced by the intervention.

The primary aim is to investigate whether positive parenting feedback, based on the “micro” intervention, has more effect on the hypothesized physiologically susceptible subgroup of parents, and to investigate why this may be the case. In what follows, we articulate the empirical basis of each of three distinct hypotheses—on parental self-efficacy beliefs, reported parenting behavior (e.g., rejection and affection) and reported child behavior (child externalizing behavior), as well as on observed parenting behavior (e.g., withdrawal and positive affect) and observed child behavior (e.g., irritability and persistence/enthusiasm)—that we will evaluate before delineating our methodology.

### Research questions/hypotheses

1. Does the randomized controlled microtrial enhance more positive parenting behavior and reduce more child externalizing behavior in “micro” intervention group compared to control condition?
  *H1*: *We expect that the “micro” intervention will bring about an increase in parents’ self-perceived competence*, *leading to behavior changes in the participating parents and children*. *We expect that immediate positive parenting feedback*, *central to successful parenting interventions*, *will increase the likelihood that parenting improves following intervention*, *as evidenced by a main effect of the intervention vs*. *control group*.
  Also, we expect that the intervention effect on parenting behavior is more pronounced when parents increase more rather than less in self-efficacy beliefs.
2. Do differential effects of responsiveness to the randomized microtrial vary as a function of parents’ and children’s physiological reactivity?
  *H2*: *We expect that some parents will disproportionally benefit from the “micro” intervention based on their physiological reactivity*, *such that those who benefit most will score higher on skin conductance and/or heart rate*, *with the same being true of their children*.
3. Do parents and children who share a high physiological reactivity prove most susceptible to the “micro” parenting intervention?
  *H3*: *We expect that those parents and children—in the same family****—****who both manifest these physiological attributes will prove most susceptible to the beneficial effects of the intervention*. *That is*, *the greatest improvement in child externalizing behavior will occur when both parent and child score high on physiological reactivity*.

## Materials and methods

### Design

The Parfective Microtrial is a randomized controlled microtrial with a “micro” intervention (i.e., immediate parenting positive feedback) and a control condition that tests physiologically-based differential susceptibility to changes in parenting. Participants are 120 families, with children aged 4–5 years, recruited form a community. Of those families, 60 will be randomly assigned to the “micro” intervention condition and 60 families to the control condition. After enrollment in the microtrial and randomization, the baseline assessment (pretest) will be carried out. The “micro” parenting intervention will be implemented after these baseline assessments. Participants in the care-as-usual control condition receive no immediate positive parenting feedback but will also be taken aside without their child, receiving only the instructions of the experiment. For the intervention condition, we will use an established parental self-efficacy microtrial intervention, which has been found to be effective in fostering parents’ positive parenting (ES = 0.63) and children’s positive behavior (ES = 0.64), by providing *immediate* positive feedback about one’s competence as a parent [10, 11]. Posttest and follow-up assessments will be conducted after 2 weeks and 4 weeks, respectively. The study procedure has been approved by the Ethics Review Board of Tilburg University.

### Recruitment

**Fig 1:**
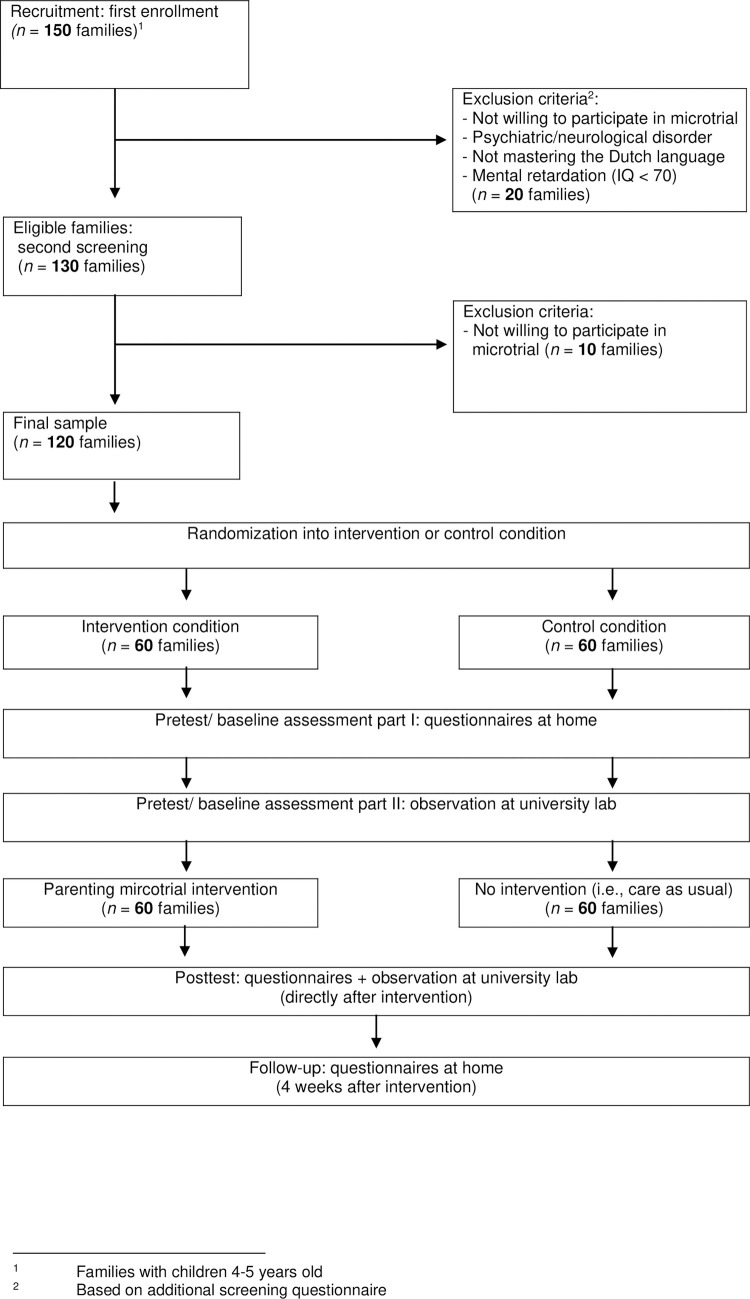
Study design (CONSORT schedule). Flowchart of the different conditions of the Parfective Microtrial.

Based on previous work that demonstrated medium effects (*d*) of the “micro” parenting intervention [10, 11, 29, 30] and a priori power analyses for investigating our hypotheses in a two-sided test at α = .05 and power (1-β) = .80 [31], data will be collected from 120 four-to-six-year-old children and their parents—recruited via flyers, social networks and letters distributed by elementary schools in The Netherlands (Fig 1). Exclusion criteria for the children will be *(a)* psychiatric/neurological disorder (as reported by the parent), *(b)* mental retardation (IQ < 70), *(c)* not mastering the Dutch language, and *(d)* that their child is not living in another household during the weekdays. The sample will be recruited, and all data collection will be carried out by the Lifespan Lab of Developmental Psychology at Tilburg University.

### Randomization

In this randomized, single-blinded, study on intervention heterogeneity, participants will be blinded to details until the end of the microtrial. Randomization (1:1) will occur through random selection of a participant number, linked to either the intervention or control condition. Participants will give consent prior to randomization (see Ethical clearance).

### Data collection/procedures

**Fig 2:**
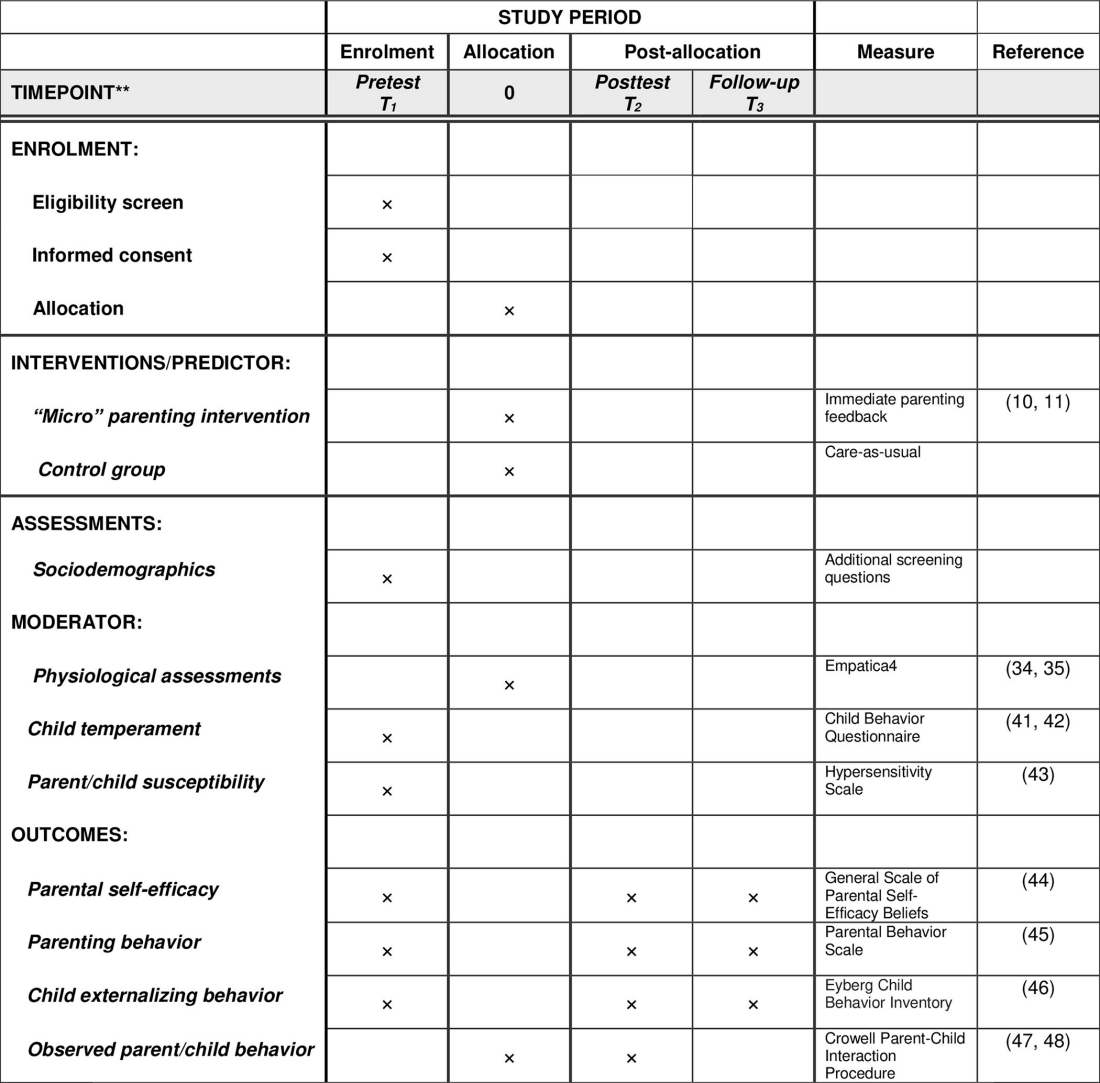
SPIRIT schedule of enrolment, interventions, and assessments. Schematic representation of measures per timepoint.^a^ T1 –Pretest.^b^ T2 –Posttest (2 weeks after baseline).^c^ T3 –Follow-up (6 weeks after baseline).

An overview of all measures and measurement occasions is given in Fig 2. All measures are widely used and found to be reliable and valid in terms of psychometric properties. Both the recruitment, as well as the data collection will be conducted in two separate cohorts in 2022/2023 and 2023/2024. The recruitment and pretest will start in October 2022 and 2023. Parents and children will be instructed to wear an Empatica E4 wristband at the university lab to measure their physiological reactivity, while receiving a 5–10-minute resting baseline session before the microtrial starts. Prior to the lab visit parents will be mailed questionnaires about their parenting practices, child behavior and environmental susceptibility. Parents will be instructed to bring the completed questionnaires with them to the university lab. Then, in the lab, parents will be observed interacting with their child. Following these baseline conditions, half of the parents will receive the “micro” parenting intervention—in the form of individual positive feedback concerning their parenting and their child’s behavior. Thereafter, at the same lab-session, parenting behavior will be observed again using the same interaction paradigm, after which we will re-administer to parents the questionnaires about their parenting and child behavior. Four weeks after the intervention we will re-administer the questionnaires about parenting and child behavior for the last time as follow-up assessment.

### Predictor

#### “Micro” parenting intervention

The experimental condition (i.e., “micro” parenting intervention vs. control) serves as predictor. All parents will come to the university lab with their child, while only half of them (1:1) receive individualized positive feedback concerning both their child and their parenting skills based on answers provided on the questionnaires completed at home. This approach has been used in other work, following ethics’ approval, and found it to be acceptable to parents—once they have been debriefed, as they will be about it at the end of the experiment. Specifically, an experimenter who self describes him/herself as a university researcher specializing in parent and child behavior will use a graph displaying “pseudo data” to compare what parents reported with responses provided by a non-existent group of parents; this presentation is designed to make parent and child look like they are doing well relative to others. Since self-efficacy beliefs (SEB) are based both on a self-evaluation that parents make about their skills and on their perception of their ability to positively influence their children [32, 33], the experimenter will emphasize the child’s positive development. All parents will be told the exact same thing: *“On the basis of the questionnaire filled out at home*, *we see that the way you behave with your child is particularly interesting for our research*. *We have noticed in earlier research we carried out that some childrearing practices*, *like yours*, *seem to be more effective in daily life*. *Based on an average calculated in earlier research with similar families in The Netherlands*, *we conclude that you are among the 20% of parents who seem to be the most effective with their children in three fields in particular*: *limit setting*, *emotion regulation and warmth”*.

The “micro” parenting intervention has been proven effective in two separate studies in fostering parents’ positive parenting and children’s positive behavior. Results showed that parents receiving positive feedback evinced more positive parenting (e.g., more positive affect towards their child) than non-reinforced parents in the control group; experimental children were more positive with their mothers (e.g., positive affect, enthusiasm towards task).

### Moderators

#### Physiological assessments

The Empatica E4 wristband will be used to measure obtain physiological signals (see https://www.empatica.com/en-eu/research/e4/). Empatica offers physiological signals in raw data format (e.g., EDA, blood volume pulse, temperature and movement) or processed format (e.g., Heart rate and inter beat interval), and offers a visualization feature [34, 35]. *EDA* (i.e, skin conduction) from the Empatica E4 will be measured with dry electrodes that detect changes in the electrical conductivity of the skin. *HR* (i.e, Heart Rate) will be recorded with a photo plethysmography (PPG) sensor that emits green (i.e., light absorption is higher in oxygenated blood) and red light (i.e., to detect motion artifacts) and samples at a frequency of 64 Hz with which a blood volume pulse (BVP) signal is obtained [36, 37]. The resolution of the sensor output is 0.9nW per digit. PPG sensors are used to measure volume changes in the blood. The PPG sensor provides a signal that can be used to calculate HR, but can also be used to calculate other vital signs such as breathing rate and blood pressure [38]. The Empatica E4 also contains an optical infrared skin *temperature* sensor that samples at a frequency of 32 Hz and a resolution of 0.02 Celsius degrees. *Movement* with the Empatica E4 will be sampled at a frequency of 32 Hz (and has a resolution of 8 bits) with a sensor that measures acceleration in space over time on an x, y, and z-axis [39].

We will use an R package software called “Wearables” and an accompanying R Shiny application called “E4 dashboard”. This software can be used by clinicians and researchers to visualize and (batch) analyze physiological signals that are obtained with the Empatica E4. To our knowledge, this is the first R package with accompanying Shiny application that simultaneously processes physiological signal data, offers flexible signal pre-processing, artifact detection, feature extraction [40], and has a built-in visualization tool.

Both parents and their children will be instructed to wear the Empatica E4 on their nondominant hand during the experiment. Prior to the microtrial intervention, they will undergo a 5-minute resting baseline session, which is a common procedure to achieve a measurement of baseline recordings to which to compare the parameters retrieved during the experiment.

### Primary outcomes

#### Parental self-efficacy beliefs

Parents’ self-efficacy beliefs (SEBs) will be assessed with the General Scale of Parental Self-Efficacy Beliefs (GSPSEB) at pretest, posttest and follow-up [41]. This 25-item scale is related to five domain-specific SEB factors: Discipline, Nurturance, Playing, Instrumental Care, and Teaching. Self-efficacy beliefs in parenting can be evaluated as a quantitative construct by asking parents their beliefs in specific parenting activities, such as teaching, playing, providing instrumental care, nurturing or disciplining their child. Items are in the form of affirmatives, for example: ‘‘I am able to sense when my child is starting to become distressed” for the Nurturance subscale. The items will be rated on a 5-point scale (1 = strongly disagree to 5 = strongly agree).

#### Reported and observed parenting behavior

Reported parenting behavior will be assessed with the short version of the Parental Behavior Scale (PBS-S) at pretest, posttest and follow-up [42]. This questionnaire consists of 25 items and comprises five subscales: Positive parenting (e.g., “I make time to listen to my child, when he/she wants to tell me something”), Discipline (e.g., “When my child has been disobedient, I give him/her a chore as punishment”), Harsh Punishment (e.g., “I spank my child when he/she is disobedient or naughty”), Material Rewarding (e.g., “I give my child candy as a reward for good behavior”), and Rule Setting (e.g., “I teach my child to be polite at school”). A 5-point scale is provided for each item, ranging from 1 = (almost) never to 5 = (almost) always.

The Crowell Parent-Child interaction task procedure will be used to observe and rate parent behavior [43, 44]. Based on the Crowell procedure, the observation task takes 20 minutes to complete and consists of four episodes: warm-up, free play, frustration task, and recovery time. The parent scales are each internally consistent and well-defined conceptually. High internal consistency for each scale provides support for the reliability of the rating scale, and suggests that the Crowell scores can be useful as two separate scales measuring a child’s affective presentation and caregiver responsiveness, but also collectively as a total score assessing overall relational functioning (e.g., [45]).

### Secondary outcomes

#### Reported and observed child behavior

One subscale assessing the degree to which child externalizing problem is a problem from the 36-item Eyberg Child Behavior Inventory (ECBI) will be used at pretest, posttest and follow-up—to assess the occurrence of reported child externalizing problem behavior [46]. This “Intensity” subscale measures frequency of externalizing behavior in children aged 2 to 16 years and has been shown to be a reliable (screening) instrument [3]. Example items are: “Does not obey house rules” and “Whines.”

Child behavior will be observed using the same interaction paradigm used to observe parent behavior [43, 44]. The parent and child scales of the Crowell procedure are each internally consistent and well-defined conceptually. As previously mentioned, high internal consistency for each scale provides support for the reliability of the rating scale, and suggests that the Crowell scores can be useful as two separate scales measuring a child’s affective presentation and caregiver responsiveness (e.g., [45]).

### Discontinuation and withdrawal

All participants are allowed—and, in case needed, are assisted–to seek mental health care and parenting support through regular services. Also, enrolled participants can withdraw from the microtrial without further explanation at any point. There will be a debriefing at the end of the experiment, where they will be thanked for her/his participation and remunerated for their participation and travel expenses. We will offer participants the possibility to contact us (or a similar institution, counsellor, or experienced researcher for data collections outside Tilburg University) in case their participation in the study was in any case emotionally stressful and they feel like they need support or want to discuss this.

### Analyses

The data will be collected anonymously by assigning an ID number. Statistical analysis will involve several steps. First, using independent t-tests and/or alternative non-parametric tests, we will examine as a preliminary analysis whether randomization was successful, comparing baseline levels of demographics, parenting behavior and child externalizing behavior across the intervention and control condition. Possible significant differences at baseline will be used as covariates in analyses [47]. Latent change (LC) models in Mplus [48] will be used to test hypothesis H1-H3 on each dependent variable—parental self-efficacy beliefs, parenting behavior, child externalizing behavior and observed parenting and child behavior [49]. Because change is measured directly through latent difference variables, representing inter-individual differences in models are an excellent approach to examining repeated measures to estimate amount of change across time. In an LC model of parenting behavior and child behavior, behavioral change is cast as a latent variable, thereby separating measurement error. The mean of the latent difference variable is used as measure of mean differences between measurements, one to be predicted by other variables, including experimental condition (i.e., “micro” parenting intervention vs. control) and physiological scores. The Bonferroni correction is used to reduce chances of obtaining false-positive results (type I errors) [50].

### Hypothesis 1

◾ The feature of the LC model which evaluates this hypothesis links the “micro” parenting intervention (vs. control) with intervention responsiveness across pretest, posttest, and follow-up assessments, Specifically, the first model affords evaluation of group differences on the change from pretest to posttest and from posttest to follow-up. The second model affords evaluation of group differences from pretest to follow-up and from posttest to follow-up. Because the “micro” parenting intervention is focused on inducing parental self-efficacy beliefs, presumed to affect parents and children by changing their competence as a parent, we will also evaluate whether intervention effects are more pronounced when parents increase more rather than less in self-efficacy beliefs. We have four outcome measurements: reported and observed parenting change and reported and observed change in child behavior.

### Hypothesis 2

◾ The same LC approach will be employed here, as delineated in H1, with the “micro” parenting intervention once again serving as the predictor in a primary model, but this time adding parents’ physiological profile and its interaction with intervention—in which each moderator will be tested separately, in separate models.
  This model controls for the correlation between parents’ physiology and pretest parenting behavior. In a second phase of modeling, a composite moderator index of physiological assessments will be created, with the same being done with their children.

### Hypothesis 3

◾ The very same LC model, as delineated in H1 and H2 will be used, but this time to evaluate the predictive value of the interaction between “micro” parenting intervention, parents’ physiology, and child’s physiology on change in child behavior. This model controls for the correlation between parents’ physiology and pretest parent behavior, between child’s physiology and pretest child behavior, between parents’ physiology and child’s physiology, as well as prior levels of child behavior.

### Ethical clearance

All procedures performed in the study involving human participants are in accordance with the ethical standards of the institutional and/or national research committee and with the 1964 Helsinki declaration and its later amendments or comparable ethical standards. The study procedure has been approved by the Ethics Review Board of Tilburg University and is registered at ClinicalTrials.gov with identification number NCT05539170. All parents will provide a structured, written, informed consent for themselves and their children. Any changes to the protocol will be registered and communicated to researchers and participants.

## Discussion

Even though parenting interventions are considered as effective treatments, the effects reported in meta-analyses are quite heterogeneous [51]. What works for whom, and why? That is an unresolved and crucial issue in parent training and therapeutic intervention more generally. More insight into parents’ differential responses and differential effectiveness of parenting interventions—in terms of affecting both parenting and child behavior—has important implications for family (and childcare) policy. A better fit between target group and intervention will result in a more efficient and effective use of resources for parent training. This approach will enable us to evaluate differential parental responsiveness to a brief and effective parenting intervention, using a randomized microtrial approach. However, as most studies on differential susceptibility to parenting that have been conducted, we used a between-subjects designs, meaning that the effects of a positive influence were assessed, but compared across susceptibility groups and not in terms of the magnitude of change in positive and negative outcome measures within each person. As such, our design does not allow to answer the specific research question of whether the same parent who profits most from immediate positive feedback would also suffer most from negative feedback. Specifically, it is an ethical issue to have participants undergo a negative environmental stimuli, to assess the “for-worse” end of the differential susceptibility spectrum [52].

### Trial status

The protocol of this study is registered at ClinicalTrials.gov with identification number NCT05539170 on September 14^th^, 2022. Protocol version 3, date 11.11.2020. The first participant has not yet been recruited. Additional details about the trial registration are presented Table 1.

**Table 1:** Overview of measurements.

Data category	Information
Primary registry and trial identifying number	ClinicalTrials.gov
	NCT05539170
Date of registration in primary registry	September 14^th^, 2022
Secondary identifying numbers	TSB_RP604
Contact for public queries	Rabia R. Chhangur e-mail: r.r.chhangur@tilburguniverisity.edu
Contact for scientific queries	Rabia R. Chhangur e-mail: r.r.chhangur@tilburguniverisity.edu
	Tilburg University, Tilburg, The Netherlands
Scientific title	Parents’ Differential Susceptibility to a Randomized Controlled Microtrial: The Role of Physiological Signals as Underlying Mechanism
Countries of recruitment	The Netherlands
Health condition(s) or problem (s) studied	Positive and negative parenting behavior and child behavior, parent-child interactions
Intervention(s)	Active comparator: “Micro” parenting intervention consisting of immediate positive parenting feedback
	Control group: Care-as-usual control condition
Key inclusion and exclusion criteria	Ages eligible for study: child, adult, older adult
	Sexes eligible for study: both
	Accepts healthy volunteers: yes
	Inclusion criteria: parents with children aged 4-years
	Exclusion criteria: psychiatric/neurological disorder (as reported by the parent), mental retardation (IQ < 70), not mastering the Dutch language, and that their child is not living in another household during the weekdays
Study type	Interventional
	Allocation: randomized intervention model, 1:1 assignment
	Masking: single (participant)
	Primary purpose: other
	Phase: not applicable
Estimated study start date	October 2022
Estimated primary completion date	August 2024
Target sample size	120
Recruitment status	Not yet recruiting
Primary outcome(s)	General Scale of Parental Self-Efficacy Beliefs (GSPSB)
	Parental Behavior Scale (PBS)
	Crowell Parent-Child Interaction Procedure: observed parenting behavior
Secondary outcome(s)	Eyberg Child Behavior Inventory (ECBI)
	Crowell Parent-Child Interaction Procedure: observed child behavior

## Supporting information

S1 File SPIRIT checklist. (PDF)

S2 File Submitted research proposal for ethics review, information letter and consent form in Dutch and English. (PDF)
