# Supplementary material for: Parents’ differential susceptibility to a “micro” parenting intervention: Rationale and study protocol for a randomized controlled microtrial
Source: PLoS One. 2023 Mar 22;18(3):e0282207. doi: 10.1371/journal.pone.0282207 (PMC10032527; doi:10.1371/journal.pone.0282207)
Supplement: S2 File — (PDF) [file pone.0282207.s002.pdf]

The application form: **G.E.D. Started!** (GDPR, Ethics, and Data Management): TBS Ethics Review Board – Case: TBS_RP604

Personal Information

School:
Which school do you want to submit your application to?
TBS

Department
Which Department do you work for?
Development Psychology

Information principal investigator
Please select your title(s)
☐ Prof. ☐ Ir.
☑ Dr. ☐ Mr.

Project duration
Please indicate the proposed start date and proposed end data of the project.
01-09-2022 - 31-08-2024

Funding organization
Is there a funding organization?
☑ There are no funding organizations
☐ The funding organizations are:

Student research
Is this research project conducted by students?
☑ No

Language
Are all documents in English?
☑ Yes

Which TiU researcher holding a PhD is responsible for this application?
Rabia Chhangur

General Information

Title

Parents' Differential Susceptibility to a Randomized Microtrial: The Role of Physiological Signals as Underlying Mechanism

Background

Parenting is widely regarded as an important *and* modifiable determinant of children's problem behavior, one which has thus proven responsive to intervention (McCart et al., 2006). While intervention effects are small in magnitude, with even the most successful programs proving effective for only 25% of enrolled families (Van Aar et al., 2017), it remains under appreciated that some parents appear to be more responsive to interventions to foster skilled parenting than others. Thus, what remains unclear is *why* some parents prove more responsive when it comes to changing their parenting in response to interventions than others. We suspect it is due, at least in part, to their own underlying physiological processes which can be measured with physiological signals (e.g., heart rate, skin conductance) by means of wearable technology.

Within the last decades, the differential susceptibility hypothesis (Belsky, 1997), a within-person phenomenon that states that the same children who are most vulnerable to adversity may also benefit most from enriched environments, has received much scientific attention. The findings seem to indicate that children differ in the extent to which they benefit from improved parenting or suffer from dysfunctional parenting due to individual differences (Bakermans-Kranenburg & Van IJzendoorn, 2015). These individual differences "for better and for worse" are believed to be rooted in certain endogenous, biological factors that include among others *(a)* temperamental characteristics and *(b)* physiological endophenotypes (Belsky & Pluess, 2009; 2013; Boyce & Ellis, 2005). So, while the experimental evidence indicates that some *children* are likely to benefit from a parenting intervention (and other environmental influences), what remains unclear is why the parenting interventions in question prove more effective in changing the behavior of some parents more than others. Could it be as a result of *parental* temperamental/physiological endophenotypes? Quite conceivably, then, some children—based on their own endophenotypes—might change more in response to change in parents' positive parenting, which itself is a result of parents' susceptibly. A possibility we aim to evaluate in the proposed research by using a randomized microtrial approach.

Endophenotypes seem to be of relevance to differential susceptibility research, because they are intrinsic traits that can be asssessed as measures of sensitivity to the environment. More specifically, physiological endophenotypic signals such

as heart rate (HR) and skin conductance (electrodermal activity, EDA) are increasingly being used in clinical practice and child healthcare research as they provide innovative ways to bring neuroscience from the lab to real-life settings (Johnson & Picard, 2020). Thus, we will rely on a very promising state-of-the-art strategy but *minimally invasive*approach, which makes it possible to evaluate differential parental/child responsiveness to a brief and effective parenting manipulation.

Although randomized trials of full-scale interventions can provide some insight into these questions, their utility is constrained by the considerable cost and effort required, and by the complex and multifaceted nature of the interventions employed. By contrast, experimental studies designed as randomized microtrials are better suited for analyzing specific effects of distinct parenting variables on parenting and child outcomes. Microtrials are based on relatively brief and focused environmental manipulations designed to suppress specific risk mechanisms or enhance specific protective mechanisms, but not to bring about full treatment or prevention effects in distal outcomes (Howe et al., 2010).

Research question(s)

1. Does the randomized microtrial enhance more positive parenting behavior and reduce more child externalizing problem behavior in intervention group compared to control condition?
2. Do differential effects of responsiveness to the randomized microtrial vary as a function of parents' physiological reactivity/temperamental characteristics?
3. Do children with a high physiological reactivity/high temperamental profile change most in behavior when their parents share their physiological/temperamental characteristics?

Study design and methodology

The project will involve a randomized-controlled experimental design. Parents will be randomly assigned to receive either a "micro" parenting manipulation to foster positive parenting and, thereby, reduce negative child behavior (n = 60) or to participate in a care-as-usual control group (n = 60) at the university lab. Those in the control group receive no feedback but will also be taken aside without their child to receive instructions about the experiment. We will use an established parental self-efficacy microtrial intervention, which has been found to be effective in fostering parents' positive parenting (ES = 0.63) and children's positive behavior (ES = 0.64), by providing *immediate* positive feedback about one's competence as a parent (Loop & Roskam, 2016; Mouton & Roskam, 2015). Our randomized microtrial manipulation/intervention is administered in a single,

20-minute session during which parents receive positive feedback regarding their positive parenting behavior and their child's positive behavior.

Procedures and materials

The sample will be recruited and all data collection will be carried out by the Lifespan Lab of Developmental Psychology at Tilburg University in The Netherlands. Families will be recruited via flyers, social networks and letters distributed by elementary schools in The Netherlands. Parents and children will be instructed to wear an Empatica E4 wristband at the lab to measure their physiological reactivity, while receiving a 5–10-minute resting baseline session before the microtrial starts. Prior to the lab visit parents will be mailed questionnaires about their parenting practices, child behavior and environmental sensitivity. Parents will be instructed to bring the completed questionnaires with them to the university lab. Then, in the lab, parents will be observed interacting with their child. Following these baseline measurements, half of the parents will receive the microtrial intervention—in the form of individual positive feedback concerning their parenting and their child's behavior. Thereafter, at the same lab-session, parenting behavior will be observed again using the same interaction paradigm, after which we will re-administer to parents the questionnaires about their parenting and child behavior. Four weeks after the intervention we will re-administer the questionnaires about parenting and child behavior for the last time as follow-up assessment.

Parenting "micro" manipulation/intervention

Parents will come to the university lab with their child and receive individualized positive feedback concerning both their child and their parenting skills based on answers provided on the questionnaires completed at home. Specifically, an experimenter who self describes him/herself as a university researcher specializing in parent and child behavior will use a graph displaying "pseudo data" to compare what parents reported with responses provided by a non-existent group of parents; this presentation is designed to make parent and child look like they are doing well relative to others. We have used this approach in other work, following ethics' approval, and found it to be acceptable to parents—once they have been debriefed, as they will be about it at the end of the experiment.

Other materials/assessments

The *Empatica E4* wristband will be used to measure obtain physiological signals and offers physiological signals in raw data format (e.g., EDA, blood volume pulse,

temperature and movement) or processed format (e.g., Heart rate and inter beat interval).

Parenting behavior will be assessed with Parental Behavior Scale (PBS-S; Van Leeuwen et al., 2018) and consists of 25 items. *Parents' self-efficacy beliefs* (SEBs) will be assessed with the Global Parental Self-Efficacy Scale (EGSCP, 2009b) and consists of 37 items. *Child externalizing behavior* will be assessed with the Eyberg Child Behavior Inventory (ECBI; Eyberg & Pincus, 1999) and *child temperament* with the *Child Behavior Questionnaire* (CBQ; Putnam & Rothbart, 2006); both consisting of 36 items. The Hypersensitivity Scale (HSC; Pluess et al., 2018) will be used to assess parents'/children's *environmental sensitivity* and consists of 12 items and the Social Information Processing Interview (SIPI-P; Ziv & Sorongon, 2011) to learn more about children's social information-processing processes.

The *Crowell Mother–child interaction task* (MCIT) procedure will used to observe and rate parent and child behavior (Crowell et al., 1991) and consists of five episodes: free play, frustration task and three increasingly difficult problem-solving puzzle tasks.

Which tools will be used for data collection and/or data analyzing?
☐ Qualtrics
☐ Mturk
☑ Qualtrics
☑ SPSS
☐ Atlas.ti
☐ R
☑ Other Mplus
☐ None

Scientific and societal relevance

Even though parenting interventions are considered as effective treatments, the effects reported in meta-analyses are heterogeneous (Mingebach, Kamp-Becker, Christiansen, & Weber, 2018). What works for whom, and why? That is an unresolved and crucial issue in parent training and therapeutic intervention more generally. More insight into parents'/children's differential responses and differential effectiveness of parenting interventions has important implications for family (and childcare) policy. A better fit between target group and intervention will result in a more effective use of resources for parent training. This approach will enable us to evaluate differential parental responsiveness to a brief and effective parenting intervention, using a randomized microtrial approach.

Have ethical issues already been assessed?
☑ No

Are you only using secondary data?
☑ No

Ethics

Population
Check the box indicating the relevant study population.
☐ Students
☑ General population without complaints
☐ General population with specific 'complaints', e.g. stress, medically unexplained complaints
☐ Patients
☐ Other

Age
Check the box indicating the age category of the participants
☑ Younger than 12 years of age
☐ Older than 11 and younger than 16 years of age
☑ 16 years or older

Recruitment
Describe the method of recruitment or selection of participants

The sample will be recruited and all data collection will be carried out by the Lifespan Lab of Developmental Psychology at Tilburg University in The Netherlands. Families will be recruited via social networks and (information) letters, distributed by elementary schools in The Netherlands, which invite them to participate in a research project on "parenting and behavior of young children".

Based on conservative estimates and prior experience (e.g., Schultz, Leijten, Shaw, & Overbeek, 2018; Zhang et al., 2020), data will be collected from 120 four-to-six-year-old children and their parents—recruited from different elementary schools and/or via flyers and social networks. Exclusion criteria for the children will be *(a)* psychiatric/neurological disorder (as reported by the parent), *(b)* mental retardation (IQ < 70), and *(c)* that their child is not living in another household during the weekdays. The sample will be recruited and all data collection will be carried out by the Lifespan Lab of Developmental Psychology at Tilburg University. Tilburg University has provided diverse

research design, sampling, data collection, data management and related services to over 300 parent-child dyads, including work with parenting manipulations/interventions, involving the collection of physiological signals.

Organization

State the organization where the recruitment of participant will take place
☑ Tilburg University
☐ Other, namely
☐ Not applicable, because

Reward

Is there a compensation for participation? If so, state what kind of compensation is offered?
☐ None
☑ Reimbursement of (travel) expenses
☐ Course credit
☑ Financial reward, namely
☑ Other, namely parent: €10 Bol.com shopping voucher; child: pack of stickers

Burden

Describe in detail the expected impact and/or potential negative consequences for the participants regarding time and mental and physical impact

The proposed study does not have any potential negative consequences (on physical and/or mental health) for participants, including parents and their children. The total duration of the experiment will be 2.5 hours, after which each participant will be debriefed and thanked for her/his participation and remunerated for their participation and travel expenses. This approach has been used in other work, following ethics' approval, and found it to be acceptable to parents—once they have been debriefed, as they will be about it at the end of the experiment.

Protection

Describe the measures that taken to protect the participants (e.g. insurance, debriefing, etc.).

The data will be administered anonymously by assigning an ID number. Also, there will be a debriefing at the end of the experiment, where they will be thanked for her/his participation and remunerated for their participation and travel expenses. We will offer participants the possibility to contact us (or a similar institution, counsellor, or experienced researcher for data collections outside Tilburg University) in case their participation in the study was in any case emotionally stressful and they feel like they need support or want to discuss this

Manipulation
Are participants subjected to procedures or experiment-related manipulations or tasks?
☑ Yes

A “micro” *manipulation* will be administered to half of the parents to foster positive parenting in a single, 20-minute session during which parents receive positive feedback regarding their positive parenting behavior and their child’s positive behavior (Loop & Roskam, 2016; Mouton, Loop, Roskam, 2018; Mouton & Roskam, 2015). Those in the care-as-usual control group will receive no feedback. Recall that randomized microtrials are defined as powerful randomized experiments, testing effects of brief environmental manipulations designed to suppress specific risk mechanisms or enhance specific protective mechanisms, but not to bring about full treatment or prevention effects on distal outcomes (Howe et al., 2010).

Mandatory documents

Information letter
Is an information letter possible?
☑ Yes

Do you use the standard TSB ERB information letter template?
☑ No

Information letter
Is an informed consent possible?
☑ Yes

Do you use the standard TSB ERB informed consent template?
☑ No

Additional documents
The following documents must be provided (if applicable):
☑ All surveys/questionnaires that will be used
☑ Description of the stimulus materials
☐ Advertisement
☐ Participants information letter (precedes participation)
☑ Written debriefing
☐ Written consent of organization(s (except Tilburg University) to recruit participants

Data storage

Data storage during research
The following questions relate to the data collection and data storage period. These are dates when all data are collected and analyzed to conduct the research.

Where will the data be stored during the data collection and data analyzing period?
☐ O-drive
☑ Research drive
☐ Surfdrive
☐ SharePoint
☐ Other

Who has access to the raw data during the data collection and data analyzing period?
Rabia Chhangur Principal investigator

Who has access to the processed data during the data collection and data analyzing period?
Rabia Chhangur Principal investigator

Data Archiving period
Data storage during data archiving. The following questions apply to data archiving. After the research is completed and the data is stored in a secure (digital) facility.

Where will the data be archived?
☑ DataVerse
☐ O-drive
☐ Other

Will you archive your data in a data repository other than DataVerse?
☑ No

Who will have access to the raw data during the data archiving period?
Rabia Chhangur Principal investigator

Who will have access to the processed data during the data archiving period?
Rabia Chhangur Principal investigator

Long-term archiving
How long will the data be archived? (In years)
10

Which criteria will you use to decide which data will have to be archived for long-term retention and access? Which (part of the) data will have to be destroyed to ensure privacy protection?

Only data needed for data underpinning a scientific publications, as well as data needed for verification purposes, including intermediate results, materials and methods of analyses along with the workflow will be archived/stored.

Should data collected during the study be stored for the required term, of 10-year period, for long-term data retention?
☑ No

Documentation and metadata
What documentation and metadata will be provided and what metadata standard will be used (if any)?

The Research Data Management (RDM) guidelines will be used for organizing data, such as (1) clear folder structure, (2) clear and consistent file names (3) clear version management (original data will be always be preserved in that it is possible to go back to earlier versions), and (4) clear organization, names and labels of variables. Also, the project will be included in the Open Science Framework (OSF).

Are you allowed to dispose of the data after the research project ends?
☑ Yes

Non-digital data
Will non-digital data be stored during the study (paper surveys, transcripts, photocopies of original documents)?
☑ No

Data sharing

Data sharing
Will all or part of the data be made available for reuse after completion of the project according to the FAIR Principles?
☑ Yes

When and how will the data be made available for re-use?

Preregistrations, syntaxes and codebooks will be shared at the Open Science Framework (OSF):

1. Each publication need to be preregistered, unless there are urgent reasons not to do so.
2. Data will be provided after preregistration.
3. Each publication will be published 100% open access, unless there are pressing reasons not to do so.
4. Covariance matrices and syntaxes will be published online (e.g., on OSF) after publication for replication purposes and meta-analyses.

To protect the privacy of participants:

1. Data will not be reproduced or transferred in any form (e.g., student-assistants are not allowed to work on data).
2. The raw data cannot be made accessible without consent of Rabia Chhangur.

Restrictions
Are there any restrictions for data sharing or any conditions for re-use of the data?
☑ No

Ownership
How will ownership of the data and intellectual property rights to the data be managed? (Explain who will be the owner of the data, meaning who will have the rights to control access.)

1. External researchers have a meeting with the team to discuss their ideas.
2. The reseachers will check whether there is no conceptual overlap between the work described in this research proposal and external researchers (as already preregistered).

3. The external researcher provides a project description, if possible beforehand.
4. The team discusses with the external researcher the feasibility of the plan (e.g., can questions be answered with the data) and potential overlap with ongoing work of researchers.
5. Iterative improvements are made until questions and hypotheses are agreed upon by each contributing researcher, and the project is considered feasible and unique compared to ongoing work.

Will there be an embargo period for (all or some of the data)?
☑ No

GDPR

Personal data collection and processing
Personal data
By completing out this part of the form you are complying with the GDPR, which requires personal data to be included in Tilburg University's data processing register. This includes a pre-DPIA (Data Protection Impact Assessment), which will reveal whether there are certain risks and whether you are obliged to perform a DPIA.

Which personal data are to be collected and processed?
☐ No personal data will be processed
☑ Yes, namely (multiple answers possible)

General
☑ Contact data (for example name, e-mail address, phone)
☑ Gender
☑ Age
☑ Birthday
☑ Nationality, birth places, birth country
☑ Experience (work education)
☑ Visual materials (pictures, video)

Special data
☑ Biometric data

Legal base
What is the lawful basis on which the processing activity takes place?
☑ (1) Consent
☐ (2) Legitimate interest as scientific researcher (*gerechtvaardigd belang)
☐ (3) Permission

Anonymization
After collection, will data be anonymized or pseudonymized? And if so, who will have access to the identifying file?

With pseudonymization, the identifying data are replaced with artificial identifiers. However, in this case, a key file with the identifying data is made, making it possible to trace them back to natural persons again.

The data will be administered anonymously by assigning a codenumber. Only Rabia Chhangur (Principal Investigator) will have acces to the identifying file. Dat will be pseudonymized because of the follow-up measurements needed to complete the study.

Processors
Are there any external parties (processors) involved in this study regarding data collection, data storage, archiving and/or other data-related activities? If so, please describe and name them here and state the website(s) of the processor(s) and/or texts.

Data collection
☑ Not applicable
☐ Yes, namely

Data storage
☑ Not applicable
☐ Yes, namely

Data archiving
☑ Not applicable
☐ Yes, namely

Other data-related activities e.g., analyses
☑ Not applicable
☐ Yes, namely

Third parties
Will you receive personal data from or provide personal data to a third party and which organization will determine the purpose for and means of the processing?
☑ No

Which third parties (data controllers and processors) are the data provided to by default?
☑ Not applicable

The Parfective Microtrial:

Over opvoeding en jonge kinderen

INFORMATIEBRIEF BIJ DE STUDIE "THE PARFECTIVE MICROTRIAL"

Deze brief is bedoeld om u verder te informeren over de studie "The Parfective Microtrial: Over opvoeding en het gedrag van jonge kinderen".

DOEL EN OPZET VAN HET ONDERZOEK

Ouders en kinderen hebben een unieke relatie die zich gedurende de eerste levensjaren ontwikkelt. Door interactie met uw kind zal deze relatie versterken. Veel ouders geven aan dat de relatie met hun kind eenvoudig en natuurlijk kan zijn, omdat ze deel uitmaakt van het dagelijkse leven, maar soms ook complex kan zijn omdat ze continue verandert en elke keer weer uniek is. Met dit onderzoek proberen we de relatie tussen ouder en kind beter te begrijpen.

Een belangrijk deel van de omgeving van jonge kinderen wordt gevormd door hun opvoeding. Kinderen kunnen verschillen in hoe gevoelig ze zijn voor interacties met hun ouders. Dit kan door allerlei factoren komen, zoals persoonlijkheid, temperament, fysiologische verschillen, of een verhoogde sensitiviteit. Maar wat weten we nu eigenlijk over individuele verschillen tussen ouders? Met dit onderzoek willen wij antwoord krijgen op de vraag waarom niet alleen kinderen maar ook ouders zo verschillend reageren op omgevingsinvloeden. Misschien herkent u dat als u denkt aan uw kinderen, of aan verschillen tussen u en uw broers of zussen.

Om meer inzicht te krijgen in de unieke relatie tussen ouders en kinderen, gaan wij 120 ouders en hun kind (tussen de 4 en 6 jaar) onderzoeken met een smartwatch die fysiologische gegevens meet. We meten in het bijzonder fysiologische activiteit, zoals hartslag omdat die volgens het meest recente onderzoek gevoelig is voor sociale interacties en dus voor uitwisselingen tussen ouder en kind. Wij zijn hiervoor op zoek naar zowel moeders als vaders en hun kind. Ook als u moeite heeft met het gedrag en/of de relatie met uw kind, is uw ervaring heel belangrijk voor ons onderzoek.

WAT HOUDT DEELNAME AAN DEZE STUDIE IN?

Als u besluit om deel te nemen aan ons onderzoek zullen wij u een online vragenlijst opsturen via email. De vragen zullen gaan over uzelf, uw gezin, en het gedrag van uw kind. U kunt deze vragenlijsten invullen op uw telefoon, laptop of ander elektronisch apparaat. De vragenlijst duurt ongeveer 20 minuten om in te vullen.

Vervolgens komt u met uw kind naar het Lifespan Lab op de campus van Tilburg University. In het lab hebben wij een speciale ruimte ingericht voor ouders en hun kind. Tijdens het bezoek zullen wij bij u en uw kind een smartwatch omdoen. Met deze smartwatch kunnen wij fysiologische activiteit van u en uw kind meten. Om de sensoren in de smartwatch goed te laten werken, vragen wij u en uw kind om de eerste 5 á 10 minuten na het omdoen van de smartwatch rustig te blijven zitten.

Wanneer de smartwatches zijn omgedaan zullen u en uw kind samen verschillende activiteiten uitvoeren in het lab. U zult bijvoorbeeld samen spelen met speelgoed dat voor jullie klaar of samen een puzzel oplossen. U hoeft zelf geen speelgoed mee te nemen. Tijdens deze activiteiten zullen videobeelden gemaakt worden van u en uw kind. Wij gebruiken deze beelden om de interactie tussen u en uw kind en het gedrag van uw kind te observeren. Het bezoek duurt ongeveer 1,5 uur. Na afloop krijgt uw kind een klein kadootje als aandenken aan het onderzoek.

Vier weken na het bezoek zullen wij u nogmaals een online vragenlijst opsturen via de email. Na het invullen van deze vragenlijsten is het onderzoek afgelopen en ontvangt u een vergoeding van €10,-. De (hoofd)onderzoeker zal u telefonisch benaderen om meer uitleg te geven over het afgeronde onderzoek en eventuele vragen beantwoorden.

VOORDELEN, RISICO'S EN BELASTING

- Het onderzoek is op geen enkele wijze nadelig voor u of uw kind.
- Het invullen van de vragenlijsten stelt u in staat om bij uw ervaringen met uzelf en uw kind stil te staan.
- Deelname aan deze studie brengt voor u geen extra kosten mee.
- U ontvangt een leuk kadootje als aandenken voor u en uw kind.
- Uw bijdrage helpt om meer inzicht te krijgen in de unieke relatie tussen ouder en kind.
- Over uw individuele resultaten kunnen we geen uitslag geven. Wel kunnen we u de gemiddelde resultaten geven van de hele onderzoeksgroep waar u als deelnemer bij hoort.

DEELNAME

- U neemt uit vrije wil deel aan dit onderzoek en u behoudt het recht op elk moment uw deelname aan het onderzoek stop te zetten.
- Het is mogelijk dat u in de toekomst wordt uitgenodigd voor deelname aan een volgende fase van onderzoek, waarbij dan de ontwikkeling van uw kind op latere leeftijd zal worden onderzocht. Uw kunt aangegeven op het toestemmingsformulier of u eventueel benaderd wilt worden of niet.

PRIVACYBESCHERMING EN VERWERKING VAN DE GEGEVENS

- Dit onderzoek is goedgekeurd door het *Ethics Review Board* van Tilburg University.
- De persoonsgegevens die u ons verstrekt door deel te nemen aan deze studie worden verwerkt door het Departement Ontwikkelingspsychologie van de Universiteit van Tilburg, onder leiding van de verantwoordelijke onderzoeker (Dr. Rabia Chhangur). De gegevens zijn uitsluitend bedoeld voor wetenschappelijk onderzoek. De verkregen resultaten worden gepubliceerd in wetenschappelijke tijdschriften.
- De verwerking van de gegevens gebeurt steeds conform de wetgeving over de verwerking van persoonsgegevens (AVG), zodat de vertrouwelijkheid van de gegevens in elk stadium van het onderzoek gegarandeerd is. De persoonsgegevens zijn slechts toegankelijk voor de betrokken onderzoekers. Zij zijn gebonden aan een wettelijke of contractuele geheimhoudingsplicht. Op verzoek kan u bij de verantwoordelijke onderzoeker een lijst krijgen van alle betrokken personen.
- Op geen enkel moment wordt uw naam verbonden aan uw ingevulde vragenlijstdata of videobeelden; deze data worden voorzien van een unieke code. Alleen de onderzoekers kunnen traceren welke code bij welke deelnemer hoort.
- De naamloze elektronische gegevensbestanden (inclusief videobeelden en fysiologische gegevens) worden minimaal 10 jaar bewaard op de universiteit.

CONTACTPERSONEN

Voor meer informatie, inlichtingen en advies kunt u contact opnemen met:

- Dr. Rabia Chhangur, hoofdonderzoeker.
    - Tel: 013 4664734
    - Email: R.R.Chhangur@tilburguniversity.edu
- Dr. Annet Toornstra, uitvoerend onderzoeker.
    - Email. A.D.Toornstra@tilburguniversity.edu

OPMERKINGEN EN KLACHTEN

Voor eventuele opmerkingen of klachten over dit onderzoek kunt u ook contact opnemen met de "*Ethics Review Board*" van Tilburg School of Social and Behavioral Sciences via ERB@tilburguniversity.edu.

Toestemmingsformulier

The Parfective Microtrial:

Over opvoeding en jonge kinderen

GELIEVE TE LEZEN, IN TE VULLEN EN TE ONDERTEKENEN

U verklaart dat u een informatiebrief heeft ontvangen en gelezen waarin alle stappen van het onderzoek worden uitgelegd en u bent bereidt deel te nemen aan de studie met uw kind. U verklaart ook dat al uw vragen vooraf beantwoord zijn. U begrijpt dat u en uw kind op elk moment de deelname aan de studie kan stopzetten, zonder dat dit voor u en uw kind gevolgen heeft en zonder dat u een reden hoeft op te geven.
Door te tekenen geeft u ons toestemming om uw (anonieme) data te verwerken, zoals genoemd in de informatiebrief. Voor eventuele opmerkingen of klachten over dit onderzoek kunt u contact opnemen met de "*Ethics Review Board*" van Tilburg School of Social and Behavioral Sciences via ERB@tilburguniversity.edu.

DEELNEMENDE OUDER

Datum: ________________________Handtekening: ______________________________

Mijn persoonlijke gegevens zijn (in blokletters invullen alstublieft):

Naam: ____

Adres: ____

Postcode: ________________________Woonplaats: __________________________________

Telefoonnummer(s): ____

E-mailadres(sen): ____

PARTNER (INDIEN VAN TOEPASSING)

Datum: ________________________ Handtekening: ______________________________

Mijn persoonlijke gegevens zijn (in blokletters invullen alstublieft):

Relatie tot het kind: ____

Naam: ____

Adres: ____

Postcode: ________________________Woonplaats: _________________________________

Telefoonnummer(s): ____

E-mailadres(sen): ____

Uw voorkeuren

In de toekomst vinden mogelijk nog vervolgonderzoeken plaats.
Mogen we u daarvoor te zijner tijd opnieuw benaderen? ☐ **Ja** ☐ **Nee**

Mogen wij foto's en video's die gemaakt worden tijdens het onderzoek gebruiken voor...

- Onderwijs, bijvoorbeeld voor psychologie studenten ☐ **Ja** ☐ **Nee**
- Onderzoek presentaties, zoals op congressen ☐ **Ja** ☐ **Nee**
- Sociale media, zoals onze Facebook pagina ☐ **Ja** ☐ **Nee**

The Parfective Microtrial:

About parenting and young children

INFORMATION LETTER FOR THE STUDY "THE PARFECTIVE MICROTRIAL"

This letter is intended to further inform you about the study "The Parfective Microtrial: On parenting and young children".

AIM AND DESIGN OF THE RESEARCH

Parents and children have a unique relationship that develops during the first years of life. Interacting with your child will strengthen this relationship. Many parents indicate that the relationship with their child can be simple and natural because it is part of everyday life, but can also be complex because it is constantly changing and is unique every time. With this research we try to better understand the relationship between parent and child.

An important part of the environment of young children is formed by their upbringing. Children can differ in how sensitive they are to interactions with their parents. This can be due to a variety of factors, such as personality, temperament, physiological differences, or increased sensitivity. But what do we actually know about individual differences between parents? With this research we want to answer the question why not only children but also parents react so differently to environmental influences. You may recognize this when you think about your children, or the differences between you and your siblings.

To gain more insight into the unique relationship between parents and children, we will investigate 120 parents and their child (between 4 and 6 years old) with a smartwatch that measures physiological data. In particular, we measure physiological activity, such as heart rate because, according to the most recent research, it is sensitive to social interactions and thus to parent-child exchanges. We are looking for both mothers and fathers and their child. Even if you have difficulty with the behavior and/or relationship with your child, your experience is very important for our research.

WHAT DOES PARTICIPATION IN THIS STUDY MEAN?

If you decide to participate in our survey, we will send you an online questionnaire via email. The questions will be about yourself, your family, and your child's behavior. You can complete these questionnaires on your phone, laptop or other electronic device. The questionnaire takes approximately 20 minutes to complete.

Then you and your child come to the Lifespan Lab on the Tilburg University campus. In the lab we have set up a special room for parents and their child. During the visit, we will put on a smartwatch for you and your child. With this smartwatch we can measure your and your child's physiological activity. In order for the sensors in the smartwatch to work properly, we ask you and your child to sit quietly for the first 5 to 10 minutes after putting on the smartwatch.

When the smartwatches are put on, you and your child will perform various activities together in the lab. For example, you will play together with toys that are waiting for you or solve a puzzle together. You do not need to bring your own toys. During these activities, video images will be made of you and your child. We use these images to observe the interaction between you and your child and your child's behaviour. The visit takes about 1.5 hours. At the end of the study, your child will receive a small gift as a memento of the research.

Four weeks after the visit, we will send you another online questionnaire via email. After completing these questionnaires, the research is over and you will receive a compensation of €10. The (principal) researcher will contact you by telephone to provide more information about the completed research and to answer any questions you may have.

BENEFITS, RISKS AND BURDEN

- The study will in no way harm you or your child.
- Completing the questionnaires enables you to reflect on your experiences with yourself and your child.
- Participation in this study will not entail any additional costs for you.
- You will receive a nice present as a keepsake for you and your child.
- Your contribution helps to gain more insight into the unique relationship between parent and child.

- We cannot give a result about your individual results. We can, however, give you the average results of the entire research group to which you belong as a participant.

PARTICIPATION

- You voluntarily participate in this study and you reserve the right to discontinue participation in the study at any time.
- It is possible that in the future you will be invited to participate in a next phase of research, in which the development of your child will be examined at a later age. You can indicate on the consent form whether you would like to be contacted or not.

PRIVACY PROTECTION AND DATA PROCESSING

- This research has been approved by the *Ethics Review Board* of Tilburg University.
- Personal data you provide to us by participating in this study will be processed by the Department of Developmental Psychology of Tilburg University, under the direction of the responsible researcher (Dr. Rabia Chhangur). The data is intended solely for scientific research. The results obtained are published in scientific journals.
- The processing of the data always takes place in accordance with the legislation on the processing of personal data (GDPR), so that the confidentiality of the data is guaranteed at every stage of the investigation. The personal data is only accessible to the researchers involved. They are bound by a legal or contractual obligation of confidentiality. On request, you can obtain a list of all persons involved from the responsible researcher.
- At no time your name will be associated with your completed questionnaire data or video images; this data is provided with a unique code. Only the researchers can trace which code belongs to which participant.
- The nameless electronic data files (including video images and physiological data) are kept at the university for at least 10 years.

CONTACTPERSONS

For more information, inquiries and advice, please contact:

- Dr. Rabia Chhangur, principal investigator.
    - Phone: 013 4664734
    - Email: R.R.Chhangur@tilburguniversity.edu

- Dr. Annet Toornstra, executive researcher.
    - Email. A.D.Toornstra@tilburguniversity.edu

COMMENTS AND COMPLAINS

For any comments or complaints about this research, you can also contact the Tilburg School of Social and Behavioral Sciences "Ethics Review Board" at ERB@tilburguniversity.edu.

Informed consent

The Parfective Microtrial:

About parenting and young children

PLEASE READE, COMPLETE AND SIGN

You declare that you have received and read an information letter explaining all steps of the study and that you are willing to participate in the study with your child. You also declare that all your questions have been answered in advance. You understand that you and your child can stop participating in the study at any time, without consequences for you and your child and without having to give a reason.

By signing you give us permission to process your (anonymous) data, as stated in the information letter. For any comments or complaints about this research, please contact the Tilburg School of Social and Behavioral Sciences “Ethics Review Board” at ERB@tilburguniversity.edu.

PARTICIPATING PARENT

Date: ________________________Signature: ______________________________

My personal details are (enter in capital letters please):

Name: ____

Address: ____

Zip code: ________________________Place: _______________________________________

Phone number(s): ____

E-mailaddress(es): ____

PARTNER (IF APPLICABLE)

Date: _______________________ Signature: _____________________________

My personal details are (enter in capital letters please):

Relationship to the child:____

Name: ____

Address: ____

Zip code: _______________________Place: ______________________________________

Phone number(s): ____

E-mailaddress(es): ____

BOTH

Your preferences

Further studies may be conducted in the future.

May we contact you again in due course? ☐ **Yes** ☐ **No**

May we use photos and videos taken during the investigation for…

- Education, for example for psychology students ☐ **Yes** ☐ **No**
- Research presentations, such as at conferences ☐ **Yes** ☐ **No**
- Social media, such as our Facebook page ☐ **Yes** ☐ **No**
